# Physiological response and sulfur metabolism of the *V. dahliae-*infected tomato plants in tomato/potato onion companion cropping

**DOI:** 10.1038/srep36445

**Published:** 2016-11-03

**Authors:** Xuepeng Fu, Chunxia Li, Xingang Zhou, Shouwei Liu, Fengzhi Wu

**Affiliations:** 1 Department of Horticulture, Northeast Agricultural University, No. 59 Mucai Street, XiangFang District, Harbin, Heilongjiang Province, 150030 China; 2 Department of Life Science and Agroforestry, Qiqihar University, No. 42 Wenhua Street, JianHua District, Qiqihar, Heilongjiang Province, 161006 China

## Abstract

Companion cropping with potato onions (*Allium cepa* var. *agrogatum Don.*) can enhance the disease resistance of tomato plants (*Solanum lycopersicum*) to *Verticillium dahliae* infection by increasing the expressions of genes related to disease resistance. However, it is not clear how tomato plants physiologically respond to *V. dahliae* infection and what roles sulfur plays in the disease-resistance. Pot experiments were performed to examine changes in the physiology and sulfur metabolism of tomato roots infected by *V. dahliae* under the companion cropping (tomato/potato onion). The results showed that the companion cropping increased the content of total phenol, lignin and glutathione and increased the activities of peroxidase, polyphenol oxidase and phenylalanine ammonia lyase in the roots of tomato plants. RNA-seq analysis showed that the expressions of genes involved in sulfur uptake and assimilation, and the formation of sulfur-containing defense compounds (SDCs) were up-regulated in the *V. dahlia-*infected tomatoes in the companion cropping. In addition, the interactions among tomato, potato onion and *V. dahliae* induced the expression of the high- affinity sulfate transporter gene in the tomato roots. These results suggest that sulfur may play important roles in tomato disease resistance against *V. dahliae.*

Tomato Verticillium wilt, caused by *Verticillium dahliae*, is a severe soil-borne fungal disease that leads to severe economic losses in greenhouses and fields. Several management strategies have been developed for controlling this disease, such as cultivating the cultivars with *Ve* gene^1^, inducing plant resistance by using beneficial bacteria^2^,^3^, intercropping^4^,^5^,^6^, and enhancing plant defense against *V. dahliae* by using sulfur^7^,^8^. These strategies would lower production costs and reduce environmental pollution. Several studies have demonstrated that intercropping and companion cropping may alleviate soil-borne diseases by enhancing plant disease resistance to pathogens^4^,^5^,^9^,^10^. Previous studies have shown that companion cropping with potato onions alleviates tomato Verticillium Wilt by up-regulating the expression levels of genes related to disease resistance (encoding proteins such as pathogenesis-related proteins and those involved in lignin biosynthesis, phytohormone metabolism and signal transduction) in tomatoes infected with *V. dahliae*^5^, and by the inhibition effect of tomato root exudates on *V. dahliae*. However, the physiological responses of tomato to *V. dahliae* infection in the tomato/potato onion companion cropping system remain unknown.

Sulfur is an essential macronutrient for plants, and, importantly, it may enhance disease defenses in plants through the formation of sulfur-containing defense compounds (SDCs), such as glutathione, glutathione-S-transferase, phytochelatins and other sulfur-containing proteins^11^. Studies have shown that the accumulations of SDCs in pathogen-resistant cultivars are rapidly increased compared with that in susceptible ones. Notably, S^0^ is the only inorganic phytoalexin reported in the available literature^12^,^13^, and Cooper *et al*. have observed higher S^0^ accumulation in the resistant genotypes of *Theobroma cacao* infected with vascular-invading fungal pathogens than in susceptible *T. cacao* plants^12^,^13^. Moreover, the accumulation of S^0^ and glutathione rapidly increases in higher resistance tomato and pepper cultivars compared with lower resistance and susceptible plants^14^,^15^. These studies have demonstrated that sulfur plays important roles in plant disease resistance. However, additional studies are needed to determine whether sulfur is involved in the disease resistance of tomato plants against *V. dahliae* in the tomato/potato onion companion cropping system.

An increased sulfur supply increases the disease resistance of plants to pathogens and limits the spread of pathogens in plants, hence alleviating disease incidence and severity, whereas sulfur deficiency increases the susceptibility of plants to pathogens^7^,^16^,^17^,^18^. Thus, determining whether tomato/potato onion companion cropping may increase the availability of sulfur in the soil would be valuable for controlling tomato verticillium wilt. Interestingly, many studies have shown that intercropping increases soil phosphorus availability via the acidification resulting from root exudates and increases iron uptake by up-regulating the expression of genes encoding iron translocator (such as IRT gene family, AtNRAMP3, AtNRAMP4 and AtVIT1) mediated by roots interplay between different plant species^19^,^20^, thereby improving phosphorus and iron nutrition for crops. Thus, it is necessary to assess whether companion cropping may improve the availability of sulfur in the tomato rhizosphere, owing to the effects of root exudates from potato onions.

In this study, to elucidate the physiological responses of tomato plants to *V. dahliae* and determine the role of sulfur in tomato disease resistance against *V. dahliae* in the companion cropping, we examined 1) the physiological responses of tomato plant to *V. dahliae* infection, 2) the expression levels of genes related to sulfur uptake and assimilation and the formation of SDCs in tomato roots in the companion cropping by using RNA-seq, 3) changes in the expression level of the high-affinity sulfate transporter gene under different conditions by using qRT-PCR, 4) the accumulation of some SDCs in tomato roots, 5) the available sulfur content in the tomato rhizosphere, and 6) the effect of root exudates from potato onions on the available sulfur content in the soil.

## Results

### The disease symptoms

**Figure 1:**
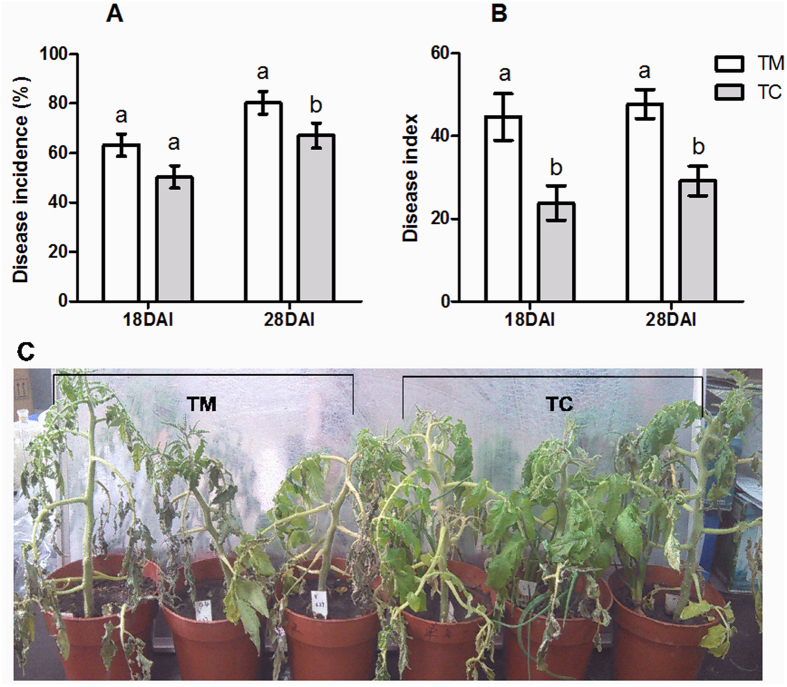
Effects of companion cropping with potato onion on the disease incidence and index of tomato verticillium wilt. TM indicates tomato monoculture; TC indicates tomato grown with potato onions. (**A**) Disease incidence. (**B**) Disease index. (**C**) Picture of disease symptom. The data are represented as the means ± SD, n = 3. The small letters above the column represent significant differences between two groups of mean values (p* *<* *0.05).

The disease symptoms of tomato verticillium wilt are presented as the disease incidence and disease index at 18 and 28 DAI (days after inoculation with Vd1). The results showed that at 18 DAI, the disease incidence was not significantly different between TM (tomatoes monoculture) and TC (tomatoes grown with potato onions) but was decreased in TC compared with that in TM (*p *<* *0.05) (Fig. 1A). In addition, the disease index was decreased in TC compared with that in TM at both 18 and 28 DAI (Fig. 1B,C).

### Changes in the content of total phenol, lignin, glutathione (GSH) and malonaldehyde (MDA) in tomato roots

**Figure 2:**
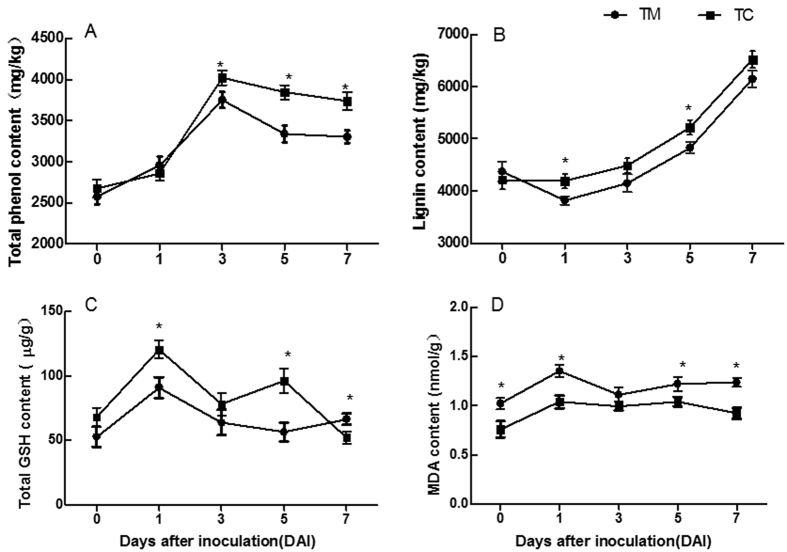
Changes in content of total phenol, lignin, GSH and MDA in the tomato roots on different days after inoculation with *V. dahliae.* (**A**) Total phenol content. (**B**) Lignin content. (**C**) GSH content. (**D**) MDA content. TM indicates tomato monoculture; TC indicates tomato grown with potato onions. The data are presented as the means ± SD, n = 3. The asterisk (*) indicates a significant difference between two groups of mean values (*p *<* *0.05).

Tomato roots were harvested at 0, 1, 3, 5, and 7 days after inoculation (DAI) with *V. dahliae* to measure the physiological responses of tomato roots to *V. dahliae* infection. The results showed that before inoculation with *V. dahliae* (0 DAI), the content of total phenol, lignin and GSH was not significantly changed between TC and TM (Fig. 2A–C), whereas the MDA content was lower in TC than in TM (*p *<* *0.05) (Fig. 2D). After inoculation with *V. dahliae,* the content of total phenol in TC significantly increased at 3, 5, and 7 DAI compared with TM, (Fig. 2A); the lignin content increased at 1 and 5 DAI (Fig. 2B); the GSH content increased at 1 and 5 DAI but decreased at 7 DAI (Fig. 2C); and the MDA content decreased at 1, 5 and 7 DAI (Fig. 2D).

### Changes in the enzymatic activities of superoxide dismutase (SOD), peroxidase (POD), polyphenol oxidase (PPO) and phenylalanine ammonia lyase (PAL)

**Figure 3:**
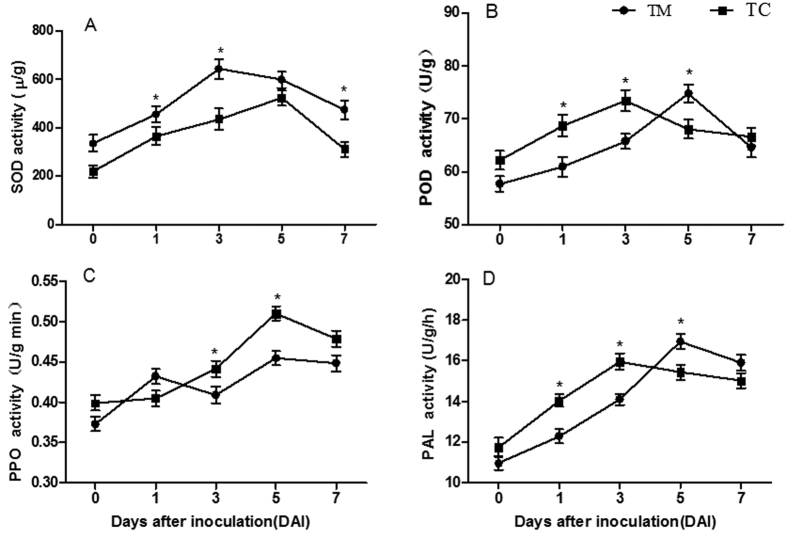
Enzyme activities of SOD, POD, PPO and PAL in tomato roots on different days after inoculation with *V. dahliae.* (**A**) SOD activity. (**B**) POD activity. (**C**) PPO activity. (**D**) PAL activity. TM indicates tomato monoculture; TC indicates tomato grown with potato onions. The data are presented as the means ± SD, n = 3. The asterisk (*) indicates a significant difference between two groups of mean values (*p *<* *0.05).

The activities of four enzymes in the tomato roots were determined at different days after inoculation with *V. dahliae.* The results showed no significant differences in the activities of the four enzymes between the two groups before inoculation with *V. dahliae* (0 DAI) (Fig. 3). At 1, 3, and 7 DAI, the SOD enzymatic activity in TC was lower than that in TM (*p *<* *0.05) (Fig. 3A). The POD activity was higher in TC at 1 and 3 DAI but was lower at 5 DAI than that in TM (Fig. 3B). The PPO activity was higher in TC at 3 and 5 DAI (Fig. 3C) than that in TM, and the trend in PAL activity was similar to that of POD (Fig. 3D).

### Expression levels of the genes related to sulfur uptake and assimilation and the formation of SDCs

**Figure 4:**
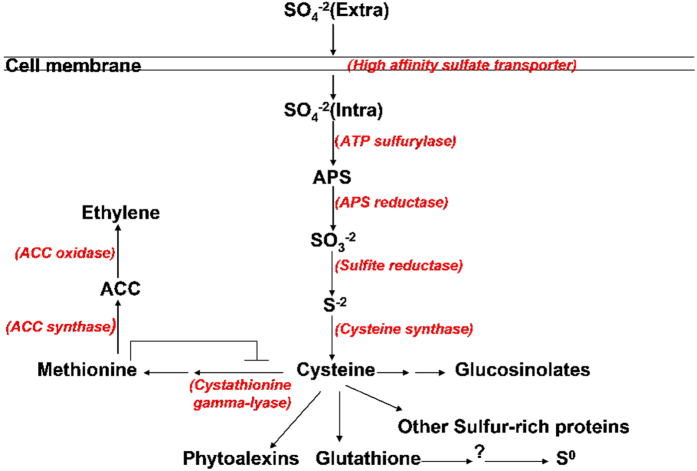
Sulfur metabolism in plants. The schematic flow diagram for sulfur metabolism, according to reviews by Thomas Rausch and Andreas Wachter^11^, and Kazu ki Saito^21^. ACC: 1-aminocyclopropane-1-carboxylate; APS: adenosine 5′- phosphosulfate; the arrow indicates induction (derepression); and the bar indicates inhibition (repression). The words in red indicate the genes encoding the enzymes (proteins) that were un-regulated in the roots of tomatoes infected by *V. dahliae* in tomato/potato onion companion cropping in the present study.

The roots of tomatoes infected by *V. dahliae* for 3 days in tomato/potato onion companion cropping were used for RNA-seq analysis. The results showed that genes involved in sulfur uptake (high-affinity sulfate transporter 2) and assimilation (sulfate adenylyltransferase and sdenylyl-sulfate reductase) and the formation of SDCs (cystathionine gamma-lyase, cysteine synthase, glutathione-S-transferase-like protein, methionine sulfoxide reductase, S-adenosylmethionine decarboxylase proenzyme and S-adenosylmethionine-dependent methyltransferase) were all up-regulated in the tomato roots in TC compared with TM under the tomato/potato onion companion cropping (Table 1, Fig. 4). The expressions of genes encoding 1-aminocyclopropane-1-carboxylate synthase and 1-aminocyclopropane-1-carboxylate oxidase, which are key enzymes catalyzing methionine to ethylene, were also up-regulated (Table 1). Notably, the expression levels of the genes encoding high-affinity sulfate transporter 2 (Solyc09g082550.2.1), glutathione-S-transferase-like protein (Solyc09g011540.2.1, Solyc01g081250.2.1), 1-aminocyclopropane-1-carboxylate oxidase (Solyc07g026650.2.1) and 1-aminocyclopropane-1-carboxylate synthase (Solyc01g095080.2.1) in TC were 10 times higher than those in TM (Table 1).

### Expression level of the high-affinity sulfate transporter 2 gene (ST2 gene)

**Figure 5:**
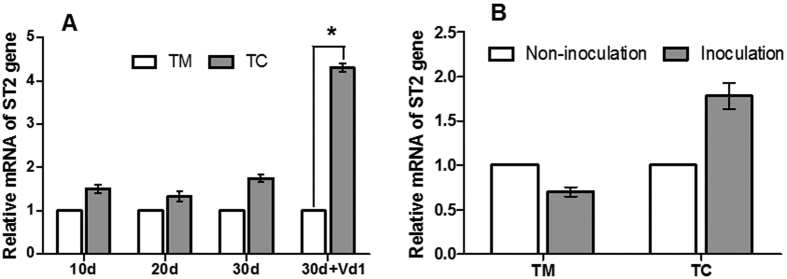
Relative mRNA expression of the ST2 gene in tomato roots infected by *V. dahliae* in tomato/potato onion companion cropping. (**A**) Effect of companion cropping with potato onions on the relative mRNA expression of the ST2 gene. (**B**) Effect of *V. dahliae* inoculation on the relative mRNA expression of the ST2 gene. TM indicates tomato monoculture; TC means tomato grown with potato onions; and 10 d, 20 d and 30 d indicate companion cropping with potato onion for 10, 20 and 30 days, respectively. 30 d + Vd1 indicates companion cropping with potato onion for 30 days with simultaneous Vd1 inoculation starting at day 20. Vd1 indicates *V. dahliae* race 1. The data are presented as the means ± SD, n = 3. The asterisk (*) indicates a significant difference between two groups of mean values (*p *<* *0.05).

The expression levels of the ST2 gene were assessed under varying conditions. The results showed that after tomato seedlings were grown with potato onion plants for 10, 20 and 30 days, the relative expression of the ST2 gene in tomato roots was not different from that in monocultured tomatoes. However, after tomato plants were grown with potato onion plants for 30 days and simultaneously inoculated with *V. dahliae* (30d + Vd1), 10 days after inoculation the relative expression of the ST2 gene in TC was 4.31 times higher than that in TM (Fig. 5A). Compared with that of un-inoculated tomatoes, the relative expression of the ST2 gene in tomatoes inoculated with *V. dahliae* was not significantly different (Fig. 5B).

### Changes in the content of total sulfur, GSH, methionine (Met) and S^0^ in tomato roots

The results showed that after inoculation with *V. dahliae* for 10 days, the content of total sulfur and GSH in the tomato roots in TC was higher than that in TM (*p *<* *0.05), whereas the Met content was lower (Table 2). S^0^ was not detected in either of the two groups.

### Changes in the content of available sulfur in the soil

**Figure 6:**
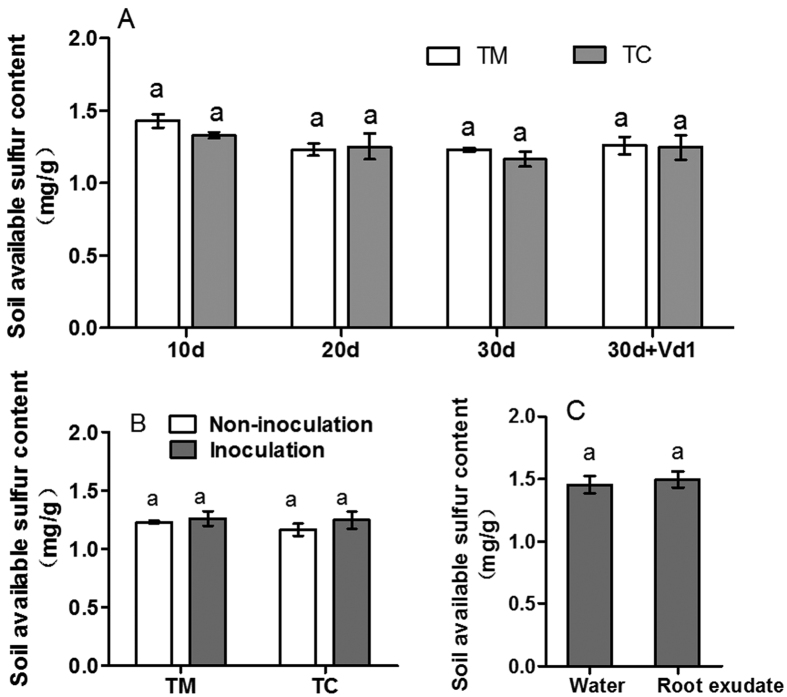
Effects of companion cropping with potato onion and potato onion root exudates on soil available sulfur content. (**A**) Effects of companion cropping with potato onion on the available sulfur content of tomato rhizosphere soil. (**B**) Effects of inoculation with *V. dahliae* on the available sulfur content of tomato rhizosphere soil. (**C**) Effects of potato onion root exudates on soil available sulfur content. The data are presented as the means ± SD, n = 3. The small letter “a” above the column represents no significance between the two groups of mean values (p > 0.05).

The results showed after the tomato plants were grown with potato onions for 10, 20 and 30 days without inoculation with *V. dahliae*, the available sulfur content in the tomato rhizosphere was not different from that in TM (Fig. 6A). After the tomato plants were grown with potato onions for 30 days and simultaneously inoculated with *V. dahliae* (30d + Vd1), 10 days after inoculation the available sulfur content in tomato rhizosphere was not different from that in TM (Fig. 6A). Compared with the un-inoculated tomatoes, tomatoes inoculated with *V. dahliae* did not show changes in the rhizosphere soil available sulfur content in either TM or TC (Fig. 6B). In addition, there was no significant difference in the available sulfur content in soil affected by the root exudates from potato onion plants (Fig. 6C).

## Discussion

The interspecific interactions of plants may enhance plant resistance to pathogens. For example, root interactions between maize and soybean increase the resistance of soybean to *Cylindrocladium parasiticum*^10^, and maize and pepper interactions enhance maize resistance to *Bipolaris maydis*^9^. Similarly, in a previous study, we have shown that tomato and potato onion interactions enhance tomato resistance to *V. dahliae* by up-regulating disease resistance-related genes under the tomato/potato onion companion cropping^5^. To explore the physiological defense responses of tomato to *V. dahliae* infection, we examined changes in the content of total phenol, lignin, GSH, and MDA, and the enzymatic activities of SOD, POD, PPO and PAL, all of which are key enzymes related to disease resistance^1^,^21^,^22^. The results showed that companion cropping with potato onions increased the contents of total phenol, lignin and GSH (Fig. 2A–C). Additional analyses showed that companion cropping with potato onions decreased the disease incidence and index (Fig. 1), confirming the results of the previous study^5^. These results indicated that companion cropping with potato onions increases the accumulation of substances related to resistance, thereby increasing the resistance of tomato plants against *V. dahliae.*

Furthermore, the activities of POD, PPO and PAL in the tomatoes grown with potato onion plants were higher than those in monocultured tomatoes at 3 days after inoculation, but POD and PAL activities in the tomatoes grown with potato onion plants were lower than those in monocultured tomatoes at 5 days after inoculation (Fig. 3B,D). These results suggested that companion cropping with potato onion plants rapidly increases enzymatic activity in the tomato roots, thereby enhancing disease resistance. In addition, POD and PAL are key enzymes in lignin biosynthesis^23^. The increased POD and PAL activities contribute to lignin accumulation, consistently with the increased lignin content in tomato roots (Fig. 2B). These results suggested that companion cropping with potato onions increases the levels and enzymatic activities of defensive substances, thereby decreasing the damage caused by *V. dahliae.* These results were confirmed by the decreased MDA content in the tomato roots (Fig. 2D), a result consistent with the decreased incidence and severity of tomato verticillium wilt (Fig. 1). Notably, the SOD activity was lower in tomatoes grown with potato onion plants than in monocultured tomatoes (Fig. 3A), probably because of enhanced pathogen defense and decreased *V. dahliae* attack^5^.

Sulfur enhances defenses against plant disease through the formation of sulfur-containing defense compounds (SDCs)^11^,^13^, and an increased sulfur supply increases the disease resistance of plants to pathogens^7^,^16^,^17^,^18^. Interestingly, the expression levels of genes related to sulfate uptake (high-affinity sulfate transporter) and assimilation and the formation of SDCs were all up-regulated in tomatoes grown with potato onion plants (Table 1), thus suggesting that sulfur-enhanced defense might play an important role in the enhanced resistance of tomatoes to *V. dahliae,* as described by Rausch and Wachter^11^. Furthermore, we examined the accumulation of SDCs, such as S^0^ and GSH, in the tomato roots. The results showed that after tomatoes were grown with potato onion plants and simultaneously inoculated with *V. dahliae*, the GSH content increased (Table 2), thus contributing to enhanced tomato resistance^11^. However, S^0^ was not detected in either of the two treatments (Table 2), possibly because S^0^ was not formed for 10 days after inoculation with *V. dahliae*. Indeed, Novo *et al*. have reported the accumulation of S^0^ at 10 days after the inoculation with *V. dahliae* in higher resistance pepper cultivars and at 15 days in lower resistance cultivars^14^. The tomato cultivar used in the present study was susceptible to *V. dahliae*, and these tomatoes were harvested at 10 days after inoculation. Nevertheless, the increased GSH content in the tomato roots (Fig. 2D, Table 2), coupled with the up-regulated expression of genes related to sulfur metabolism, such as those encoding glutathione-S-transferase, cysteine synthase and peptide methionine sulfoxide reductase, probably contribute to the enhancement of tomato resistance against *V. dahliae*.

Interestingly, RNA-seq analysis revealed that the expression level of the high-affinity sulfate transporter 2 gene (ST2), primarily responsible for sulfate uptake from the soil^22^, in tomatoes grown with potato onion plants was higher than that in monocultured tomatoes (Table 1). However, we were interested in the factors that induce the up-regulation of the ST2 gene. Therefore, we examined changes in the expression of the ST2 gene under different conditions. The results showed that after tomatoes were grown with potato onion plants without inoculation with *V. dahliae*, the expression of the ST2 gene in the intercropped tomatoes was not changed at each growth stage, as compared with that in monocultured tomatoes (Fig. 5A). However, after inoculation with *V. dahliae*, ST2 gene expression in tomatoes grown with potato onion plants was significantly higher than that in monocultured tomatoes (Fig. 5A). In addition, compared with un-inoculated tomatoes, tomatoes inoculated with *V. dahliae* did not show increased ST2 gene expression under monoculture (Fig. 5B), thus indicating that inoculation with *V. dahliae* did not induce the expression of the ST2 gene. This finding was not consistent with the result described by Howarth *et al*.^24^, in which infection with *V. dahliae* induced a high expression of LeST1-2 (one of the high-affinity sulfate transporters genes) in a resistant tomato cultivar. This discrepancy may be attributed to the susceptible tomato cultivar used in the present study. These results suggest that the up-regulated expression of the ST2 gene is induced by the interactions among all three factors—potato onion, tomato and *V. dahliae*—rather than interactions between two factors, i.e., potato onion and tomato, or tomato and *V. dahliae*.

There are two potential causes for the up-regulation of the ST2 gene in the tomato roots: sulfur-deficiency in tomato rhizosphere soils^24^,^25^ and pathogen infection^11^. Sulfur is taken up into plants as sulfate and subsequently assimilated into sulfur-containing amino acids, eventually forming SDCs^11^. Previous studies have shown that the genes encoding high-affinity sulfate transporters are up-regulated in tomato roots in response to sulfate deprivation^24^,^25^ and infection with *V. dahliae* in resistant tomato cultivars^24^. However, the results of the present study showed that inoculation with *V. dahliae* alone did not alter the expression level of the ST2 gene (Fig. 5B). In addition, we monitored the available sulfur content in the tomato rhizosphere, and the results showed that the available sulfur content in the rhizosphere was not significantly different between the groups, regardless of inoculation with *V. dahliae* (Fig. 6A,B). These results suggested that the up-regulated expression of the ST2 gene was not induced by either soil sulfate deficiency or *V. dahliae* infection alone.

In this case, we propose that a demand-driven mechanism might be responsible for the up-regulation of ST2 gene expression under infection conditions in tomato/potato onion companion cropping^11^,^25^. Plants positively respond to *V. dahliae* infection by forming more SDCs. The increased demand for SDCs requires an increase in the flux from cysteine to methionine (Met), GSH, S^0^ and other sulfur-rich proteins, and, therefore, leads to the up-regulation of the ST2 gene^11^,^25^. Feedback regulation was exhibited for the expression of the high-affinity sulfate transporter gene in response to the increased production of SDCs (Fig. 4). This mechanism was further supported by the results shown in Tables 1 and 2. Met is an intermediate in sulfur metabolism and a rate-limiting substance for sulfur-containing amino acid metabolism^25^,^26^. Met production is self-regulated through a feedback mechanism, i.e., if the Met content is sufficiently high, the production is decreased^25^. In this study, the Met content in tomatoes grown with potato onion plants was lower than that in monocultured tomatoes (Table 2). This result might reflect the high expression of genes encoding 1-aminocyclopropane-1-carboxylate oxidase and 1-aminocyclopropane-1-carboxylate synthase, which catalyze Met and form ethylene (Table 1, Fig. 4). The decreased Met level would lead to a temporary deficiency, thereby increasing cysteine consumption, which would lead to an increase in the expression of the high-affinity sulfate protein gene^25^.

Because the sulfur supply contributes to the disease resistance of plants^7^,^8^, the management of sulfur nutrition in the soil will be beneficial for plant defenses against pathogens. Several studies have demonstrated that the interactions between plants species may improve phosphorus and iron nutrition in plant rhizospheres through root exudates in intercropping systems^19^,^27^. Thus, it is important to evaluate whether the root exudates of potato onion plants may improve soil sulfur nutrition. In this study, we found that the root exudates of potato onion plants did not improve soil sulfur nutrition (Fig. 6C). However, the results suggested that companion cropping with potato onion plants increases sulfate uptake in tomato roots infected by *V. dahliae,* thereby increasing tomato resistance against *V. dahliae.* These findings might more effectively help farmers manage tomato Verticillium Wilt and reduce fungicide application and environmental pollution.

## Conclusions

Companion cropping with potato onions increased defense substances (total phenol, lignin and GSH) and enzymatic activities (POD, PPO, and PAL) under *V. dahliae* stress. In addition, companion cropping with potato onions up-regulated the expression of genes related to sulfur metabolism and increased the total sulfur and GSH content in tomato roots, thereby indicating that sulfur might play important roles in tomato disease resistance to *V. dahliae.* Companion cropping with potato onion plants and potato onion root exudates did not alter soil sulfur availability. Thus, companion cropping might be the demand-driven mechanism by which potato onion plants up-regulate the expression of the high-affinity sulfate transporter gene. This discovery demonstrates an important step in the understanding of sulfur-enhanced defense, mediated by plant-plant-pathogen interactions in tomato/potato onion companion cropping.

## Materials and Methods

### Cultivation of plant materials and preparation of the fungal spore suspension

The tomato cultivar “Qiyanaifen” (*V. dahliae*-susceptible) and potato onion cultivar “Suihua” were used in this study. The fungal pathogen, *V. dahliae* race 1 (Vd1) was also used. Tomato cultivation and the preparation of the fungal spore suspension were performed according to a previous study^5^. Briefly, tomato seedlings were cultured in a greenhouse. The fungal spore suspension, at a concentration of 1.0 × 10^7^ spores/mL, was prepared according to the methods described by Dobinson *et al*.^28^

### Experiment design and sampling

The pot experiments were conducted from April to July 2015 in a greenhouse located in the Experimental Center of Northeast Agricultural University, Harbin, China. The experiments included the following two treatments: tomato/potato onion companion cropping (TC), in which one tomato seedling was grown with two potato onion plants at a distance of 10 cm between the tomato and potato onion plants, and tomato monoculture (TM), in which one tomato seedling was grown alone (without potato onion) in a single pot. Tomato seedlings with four true leaves were transplanted into pots (20 × 7.5 × 11.5 cm) filled with 2.5 kg autoclaved field soils per pot. Autoclaved underground water was applied throughout the experiment.

All the tomato seedlings were divided into three groups for different measurements (Table 3). In the first group, 20 days after transplantation, the tomato seedlings were inoculated with 20 mL of the Vd1 spore suspension (1 × 10^7^ spores/mL) poured onto the rhizospheres of each tomato seedling. The tomato roots were harvested at 0, 1, 3, 5, and 7 days after inoculation (DAI) with *V. dahliae,* immediately frozen in liquid nitrogen after cleaning with autoclaved deionized water, subsequently stored at −80 °C for the determination of physiological changes. For each sampling, the roots from 3 tomato seedlings were collected and pooled together as one sample, with 3 samples per treatment (n = 3). For the remaining 60 tomato seedlings (10 tomato seedlings per treatment in one replication and there were 3 replications), the symptoms of Verticillium wilt disease were observed from 10 to 30 DAI at 3-day intervals. The disease symptoms were evaluated using a 0–5 scale according to a previous study^5^. The disease incidence was defined as the percentage of tomato seedlings with disease symptoms among all treated seedlings in each treatment per replication. The disease index (DI) was determined as *DI* = 100 × (∑*Si* × *Ni*)/(5 × *Ni*) according to the method described by Mercado-Blanco *et al*.^29^, where *Si* is the symptoms severity (disease scale) of an individual tomato plant, *Ni* is the number of plants with *Si* symptoms severity, and *Nt* is the total number of plants in each treatment per replication.

In the second group, 20 days after transplantation, tomato seedlings were inoculated with 20 mL of Vd1 spore suspension (1 × 10^7^ spores/mL) poured onto the tomato seedling rhizospheres. Three days after inoculation with Vd1, the roots of 5 tomato seedlings in each treatment per replicate were harvested and pooled as one sample, with 3 samples per treatment (n = 3). The samples were instantly frozen in liquid nitrogen after cleaning with autoclaved deionized water and subsequently stored at −80 °C for RNA-seq library preparation.

In the third group, 10, 20 and 30 days after transplantation, the roots of tomato seedlings were harvested, immediately frozen in liquid nitrogen after being cleaned with autoclaved deionized water, and subsequently stored at −80 °C for determining the expression levels of high-affinity sulfate transporter gene. Additionally, 20 days after transplantation, parts of the tomato seedlings per treatment were inoculated with 20 mL of Vd1 spore suspension (1 × 10^7^ spores/mL) poured onto the tomato seedling rhizospheres. 10 days after inoculation, the tomato seedlings roots and tomato rhizosphere soils were harvested and collected as above to determine the high-affinity sulfate transporter gene expression level and available sulfur content, respectively. The tomato rhizosphere soils were collected according to the method described by Wang *et al*.^30^. Briefly, the roots of tomato seedlings were carefully harvested and shaken gently to remove bulk soils. The soils adhering to the roots were treated as rhizosphere soil samples and collected using a brush. The rhizosphere soils were passed through a 2-mm mesh sieve and subsequently air-dried to measure the available sulfur content. Whole tomato plants from both treatments were harvested, cleaned, dried in an oven at 75 °C for 72 h, and subsequently mashed to determine the total sulfur content. For tomato roots and soil samples, 3 tomato seedlings and rhizosphere soil samples were pooled as single samples, in each treatment, with 3 samples per treatment in total (n = 3).

Experiments to determine the effects of potato onion root exudates on soil available sulfur content were performed according to the method described by Ae *et al*., with few modifications^31^. Briefly, 20 g of field soil was placed in a beaker, covered and autoclaved three times to eliminate soil microbes. Subsequently, 10 mL of sterile potato onion root exudates at a concentration of 1 g root/10 mL was added to the soil on a sterile operating platform at an interval of 24 h. Sterile deionized water instead of potato onion root exudates was added as a control treatment. There were 5 replicates of each treatment, and all beakers were stored in the dark at 25 °C. The soils were air-dried and passed through a 2-mm mesh sieve to determine the available sulfur content.

### Measurement of the physiological parameters

A 0.1-g root sample of the fine powder material from each treatment and replication was used to examine the total phenol and lignin content, according to the methods described by Rodrigues *et al*.^32^. Total soluble phenolics were expressed as mg of catechol per kg of root fresh weight, and total lignin was expressed as mg/kg of root fresh weight by using lignin alkali as a standard. A 0.5-g frozen root sample from each treatment and replication was ground in 2 mL of cold extraction buffer (0.05 M phosphate buffer, pH 7.8), and the crude extract was used to determine SOD, POD, PPO, and PAL activities and the MDA content according to the methods described by Wang *et al*.^33^. POD activity was estimated using the guaiacol method^34^. PPO activity was spectrophotometrically measured on the basis of the increase in colored oxidation products within the first 3 min of the reaction^35^. PAL activity was assayed on the basis of the catalyzed rate of phenylalanine, as previously described^33^. A thiobarbituric acid reaction was used to determine the MDA content^36^. The levels of total glutathione content in tomato roots extracted in 2 mL of 0.1 M sodium phosphate buffer containing 5 mM EDTA (pH 7.5) were determined on the basis of the reaction of GSH with DTNB [5,50-dithio-bis (2-nitrobenzoic acid)], which produces the yellow derivative 50-thio-2-nitrobenzoic acid (TNB), according to the procedure described by Yu *et al*.^37^ The methionine level in tomato roots was determined using an Automatic Amino Acid Analyzer (Hitachi High-Technologies Corporation, Tokyo, Japan) after hydrolysis with HCl, and an external standard was used to calculate the concentration in tomato roots^38^,^39^. An Elementary Analysis System (Vario EL Cube, Germany) was used to determine the S^0^ level in tomato roots, according to the general procedure for elemental analyzers (JY/T017-1996, China). The total sulfur content in whole tomato plants was determined on the basis of HNO_3_-HClO_4_ digestion coupled with Barium Sulfate Turbidimetry^40^,^41^. The soil available sulfur content was determined through Ca(H_2_PO_4_)_2_-NaHCO_3_ extraction coupled with Barium Sulfate Turbidimetry^42^. All the measurements were performed in triplicate.

### Screen of differentially expressed genes (DEGs) with RNA-seq

The tomato root samples were used to screen DEGs with RNA-seq 3 days after inoculation with Vd1 in tomato/potato onion companion cropping. Some of the results obtained in this study have been presented in a previous report (doi: 10.3389/fpls.2015.00726)^5^, in which we have summarized the DEGs related to disease resistance (such as pathogenesis-related protein genes, lignin biosynthesis related genes, plant hormones metabolism related genes), but the genes related to sulfur uptake and assimilation and the formation of SCDs were not listed. In this study, we presented the DEGs related to sulfur uptake and formation of SCDs from all DEGs.

### Primer design and qRT-PCR assay for the high-affinity sulfate transporter (ST2) gene

The tomato *actin* gene was used as a reference gene^43^,^44^. The gene-specific primer pairs for the ST2 and *actin* genes were designed using Primer 5.0 software (Premier, Canada) and synthesized at the Sangon Biotech Company (Shanghai, China). The primer pair sequences for the ST2 and *actin* genes were F 5′-3′CAAAATTCTTCTGGATAAGTGCTA/ R 5′-3′CAAGGCGATGATACTGGTGAC, and F 5′-3′GAAATAGCATAAGATGGCAGACG/ R 5′-3′ATACCCACCATCACACCAGTAT, respectively. Total RNA from tomato roots was extracted using the TRIzol method described by Pattemore^45^. First-strand cDNA was synthesized using the TIANScript RT Kit (TIANGEN, China), according to the manufacturer’s instructions. The cDNA concentration was determined by using an Ultramicro ultraviolet spectrophotometer. The qRT-PCR reactions were performed with an iQ5 Multicolor Real-Time PCR Detection System (BIO-RAD, USA) with RealmasterMix (SYBR Green) (TIANGEN, China) according to the manufacturer’s instructions. The reaction mixture (20 μL) contained 0.6 μL of template cDNA, 0.3 μL of each primer oligonucleotide, 9 μL of fluorescent dyes and 9.8 μL of RNase-free ddH_2_O. Sterile water was used as a negative control, and all the samples were run in triplicate. The reaction mixture was denatured at 95 °C for 5 min, and this was followed by 35 cycles at 95 °C for 50 s, annealing at 57 °C for 30 s and extension at 72 °C for 40 s. At the end of 35 cycles, an additional final extension at 72 °C for 5 min was conducted to extend any premature DNA synthesis^46^. The mRNA expression levels of the target genes were normalized relative to the expression of the tomato *actin* gene and calculated using the 2^−ΔΔCt^ method.

### Statistical analysis

Analysis of variance (ANOVA) was performed using SPSS 16.0 analysis software (SPSS Inc., USA) and Tukey’s tests at the *p* = 0.05 significance level.

## Additional Information

**How to cite this article**: Fu, X. *et al*. Physiological response and sulfur metabolism of the *V. dahliae*-infected tomato plants in tomato/potato onion companion cropping. *Sci. Rep.* **6**, 36445; doi: 10.1038/srep36445 (2016).

**Publisher’s note:** Springer Nature remains neutral with regard to jurisdictional claims in published maps and institutional affiliations.

## Figures and Tables

**Table 1:** Changes in the expression of genes related to the sulfur metabolism in tomatoes infected by *V. dahliae* in the tomato/potato onion companion cropping (n = 3).

Gene ID	Expression fold (TC/TM)	Probability	Description
Solyc09g082550.2.1	17.63	0.848035694	High affinity sulfate transporter 2
Solyc03g005260.2.1	3.59	0.858635510	Sulfate adenylyltransferase
Solyc02g032860.2.1	4.19	0.862731753	Adenylyl-sulfate reductase
Solyc08g083110.2.1	3.16	0.821302983	Cystathionine gamma-lyase
Solyc10g012370.2.1	3.34	0.846850935	Cysteine synthase
Solyc09g011620.1.1	9.13	0.896062565	Glutathione S-transferase-like protein
Solyc09g011540.2.1	13.21	0.903839125	Glutathione S-transferase-like protein
Solyc01g081250.2.1	24.54	0.902061986	Glutathione-S-transferase
Solyc03g111720.2.1	2.76	0.815851829	Peptide methionine sulfoxide reductase
Solyc02g089610.1.1	2.86	0.83312537	S-adenosylmethionine decarboxylase proenzyme
Solyc04g040180.2.1	4.69	0.871623751	S-adenosylmethionine-dependent methyltransferase
Solyc01g095080.2.1	13.46	0.894367351	1-aminocyclopropane-1-carboxylate synthase
Solyc07g026650.2.1	15.26	0.819387202	1-aminocyclopropane-1-carboxylate oxidase

Note: TM indicates tomato monoculture; TC indicates tomato grown with potato onions. The tomato roots used for the RNA-seq analysis were inoculated with *V. dahliae* 3 days before the analysis in both groups. Probability ≥ 0.8 and expression-fold (TC/TM) ≥ 2 were examined as the threshold to determine the significance of gene expression differences between the two groups.

**Table 2:** Effects of companion cropping with potato onion on the content of total sulfur, GSH, Met and S^0^ (n = 3).

Treatment	Total sulfur (mg/g)	Glutathione (μg/g)	Methionine (μg/g)	S^0^ (μg/g)
TM	4.58 ± 0.31b	28.8 ± 4.06b	1.57 ± 0.21a	n.d
TC	5.60 ± 0.32a	41.9 ± 3.76a	0.82 ± 0.27b	n.d

Note: TM indicates tomato monoculture; TC indicates tomato grown with potato onions. In both groups, tomatoes were grown for 30 days and inoculated with *V. dahliae* on day 20, and the roots were harvested 10 days later. The different small letters represent the significance between two groups of mean values at p = 0.05 according to independent sample t-tests. n.d. indicates not detectable.

**Table 3:** Treatments and arrangements for sampling and measurements (n = 3).

Measurements	Treatment	Days after transplanting*	Days after inoculation**
Physiological responses and disease symptoms	TM	20	0,1,3,5,7 and 10 to 30
	TC	20	0,1,3,5,7 and 10 to 30
RNA-seq	TM	20	3
	TC	20	3
Expression level of ST2 gene, soil available sulfur content, SDCs content (inoculation with Vd1 for 10 days)	TM	10	—
		20	—
		30	—
		30	10
	TC	10	—
		20	—
		30	—
		30	10

^*^Note: Transplanting indicates the tomato seedlings with four true leaves were transplanted into pots. On the same day, potato onions were grown with one tomato plant in the same pot, as TC; the tomato monoculture (without potato onion) is represented as TM. ** Inoculation of tomatoes with *V. dahliae* race 1 (Vd1). “—” No inoculation.
